# Two tagSNPs rs352493 and rs3760908 within *SIRT6* Gene Are Associated with the Severity of Coronary Artery Disease in a Chinese Han Population

**DOI:** 10.1155/2016/1628041

**Published:** 2016-03-28

**Authors:** Sai-sai Tang, Shun Xu, Jie Cheng, Meng-yun Cai, Lin Chen, Li-li Liang, Xi-li Yang, Can Chen, Xin-guang Liu, Xing-dong Xiong

**Affiliations:** ^1^Institute of Aging Research, Guangdong Medical University, Dongguan 523808, China; ^2^Institute of Biochemistry & Molecular Biology, Guangdong Medical University, Zhanjiang 524023, China; ^3^Key Laboratory for Medical Molecular Diagnostics of Guangdong Province, Guangdong Medical University, Dongguan 523808, China; ^4^Department of Cardiovascular Disease, The First People's Hospital of Foshan, Foshan 528000, China; ^5^Department of Cardiovascular Disease, The Affiliated Hospital of Guangdong Medical University, Zhanjiang 524023, China

## Abstract

*SIRT6 *has been demonstrated to exert protective effects on endothelial cells and is closely associated with lipid metabolism, glucose metabolism, and obesity, indicating an important role in the pathogenesis and progression of coronary artery disease (CAD). Nonetheless, the biological significance of* SIRT6* variants on CAD is far to be elucidated. Here we aimed to investigate the influence of* SIRT6* polymorphisms on individual susceptibility and severity of CAD. Multivariate logistic regression analysis exhibited no significant association between these five polymorphisms and CAD risk in the genotype and allele frequencies. However, we found that the rs352493 polymorphism in* SIRT6* exhibited a significant effect on the severity of CAD; C allele (*χ* ^2^ = 7.793, adjusted *P* = 0.013) and the combined CC/CT genotypes (*χ* ^2^ = 5.609, adjusted *P* = 0.031) presented the greater CAD severity. In addition, A allele (*χ* ^2^ = 5.208, adjusted *P* = 0.046) and AA (*χ* ^2^ = 4.842, adjusted *P* = 0.054) of rs3760908 were also associated with greater CAD severity in Chinese subjects. Our data provided the first evidence that* SIRT6 *tagSNPs rs352493 and rs3760908 play significant roles in the severity of CAD in Chinese Han subjects, which might be useful predictors of the severity of CAD.

## 1. Introduction

Coronary artery disease (CAD) poses the predominant health threat to the public and is the leading cause of death and morbidity worldwide. Previous studies and clinical trials have demonstrated that various environmental factors contribute to the development of CAD, including obesity, diabetes, alcohol intake, tobacco use, hypercholesterolemia, dyslipidemia, and hypertension [1, 2]. Moreover, apart from these modifiable risk factors, accumulating evidences have established that genetic variants or polymorphisms in candidate genes are closely associated with CAD pathogenesis, which have been estimated to account for 40–60% of the risk for CAD in epidemiology, family, and twin studies [3, 4].

*SIRT6*, one of the seven members of the NAD(+)-dependent histone deacetylase family of sirtuins, has been considered as one of the most important longevity genes [5–7]. Transgenic mice overexpressing* SIRT6* exhibit a significantly longer life span, while the* SIRT6*-deficient mice have severe metabolic defects, developed ageing-like phenotypes by 2-3 weeks of age, and eventually died at about 4 weeks [5, 8]. In addition to the life span regulation function, a growing body of evidences have unraveled that* SIRT6* also exerts crucial effects on cell metabolism and DNA damage repair by directly influencing the expression of target genes [9–13]. Among these, the association between* SIRT6* and lipid metabolism has been well established.* SIRT6* has been reported to serve as a negative regulator of TG synthesis and low-density lipoprotein (LDL) cholesterol homeostasis.* SIRT6* deficiency resulted in accumulation of TG and LDL cholesterol levels [14–16]. Vice versa,* SIRT6*-overexpressing mice exhibit a decrease in visceral fat accumulation and improved blood lipid profile [17]. Moreover, the sterol-regulatory element binding protein, a key regulator of cholesterol biosynthesis, has been validated as a substrate of SIRT6 [18].* SIRT6* was found to repress the expression of sterol-regulatory element binding protein by suppressing its promoter activity and protein cleavage and activating the AMPK signal pathway [19]. Knockout of* SIRT6* caused elevated serum cholesterol levels, while ectopic expression of* SIRT6* diminished the serum cholesterol levels, thus improving hypercholesterolemia and atherosclerosis [20, 21]. Therefore, it was reasonable to speculate that* SIRT6* might exert an important role in CAD pathogenesis and progression.

Single nucleotide polymorphisms (SNPs) are the most frequent genetic variants in the human genome, which have been demonstrated to affect individual susceptibility for a variety of human diseases. Mounting evidences have suggested that SNPs within the candidate genes may potentially contribute to the development of CAD [22–24]. Nonetheless, the genetic causes and underlying molecular mechanisms of these candidate genes for CAD pathogenesis are still far to be elucidated. As* SIRT6* plays crucial roles in cellular senescence, genome integrity, and lipid metabolism and improves hypercholesterolemia and atherosclerosis [25], we speculated that the SNPs within the* SIRT6* gene might influence the susceptibility and severity of CAD as well. Thus, we herein conducted a case-control study to investigate the associations of five tagSNPs (rs11878868, rs107251, rs352493, rs4807546, and rs3760908) in* SIRT6* gene with the risk and severity of CAD. Our results uncovered that rs352493 and rs3760908 polymorphisms within* SIRT6* gene were associated with the higher CAD severity in Chinese Han subjects.

## 2. Materials and Methods

### 2.1. Study Subjects

The study population consisted of 1129 subjects (655 CAD-free controls and 474 CAD patients). All enrolled subjects were recruited from the Affiliated Hospital of Guangdong Medical University (Zhanjiang, China) and the First People's Hospital of Foshan (Foshan, China) from March 2011 to October 2014. All CAD subjects were previously untreated and newly diagnosed. The diagnosis of CAD was confirmed by coronary angiography performed with the Judkins technique. CAD was defined as angiographic evidence of at least one segment of a major epicardial coronary artery with more than 50% organic stenosis. Patients were divided into three groups (1-vessel, 2-vessel, and 3-vessel stenosis) according to the number of significantly stenotic vessels. A total of 655 control individuals were consecutively recruited from the two hospitals for regular physical examinations when CAD subjects were recruited. The unaffected controls were defined to be free of CAD by clinical examination, medical history, electrocardiography, and questionnaires. Subjects with peripheral vascular disease, rheumatic heart disease, congestive heart failure, pulmonary heart disease, hepatic disease, chronic kidney, or any malignancy were excluded from the study. All enrolled subjects were genetically unrelated Han Chinese. The diagnosis of hypertension was established if the individual's systolic blood pressure (SBP) was above 140 mm Hg or diastolic blood pressure (DBP) was above 90 mm Hg or if the patient was on antihypertensive medication, respectively. Diabetes mellitus was defined as fasting blood glucose (FBG) being above 7.0 mmol/L or patient being on antidiabetic drug treatment. Subject was defined as smoker if he/she smoked once a day for over 1 year. Dyslipidemia was defined as triglyceride (TG) concentration being >1.70 mmol/L or total cholesterol (TC) concentration being > 5.72 mmol/L or patient being on lipid-lowering therapy. This study was approved by the Ethics Committee of the Affiliated Hospital of Guangdong Medical University and the First People's Hospital of Foshan, and informed consent was obtained from all recruited subjects. A structured questionnaire was administered to collect information on demographic data and the risk factors related to CAD.

### 2.2. Biochemical Analysis

An approximately 2 mL blood sample was collected from each participant into tubes containing EDTA. The blood sample was centrifuged at 2000 ×g for 15 min after collection and then stored at −80°C until analysis. Serum concentrations of TC, TG, low-density lipoprotein cholesterol (LDL-C), and high-density lipoprotein cholesterol (HDL-C) were measured enzymatically on a chemistry analyzer (Olympus, Japan). Glucose was measured via the glucose oxidase method (Abbott Laboratories, USA).

### 2.3. DNA Extraction

Genomic DNA was isolated from peripheral whole blood using TIANamp blood DNA extraction kit (TianGen Biotech, Beijing, China). All DNA samples were dissolved in water and then stored at −20°C until use.

### 2.4. TagSNP Selection and Genotyping

**Figure 1:**
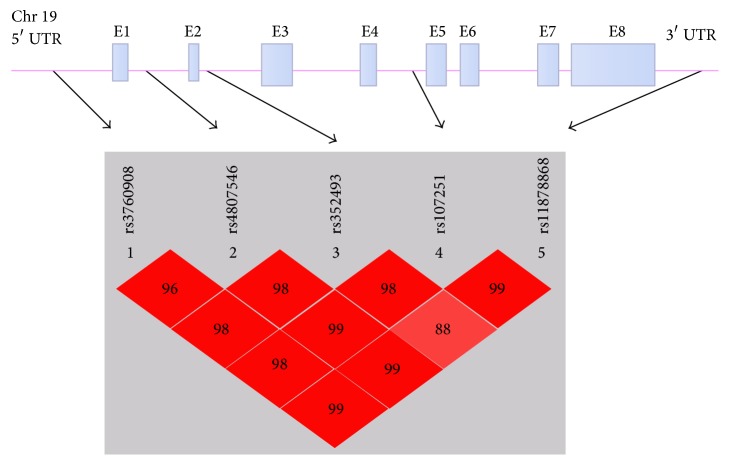
Schematic of* SIRT6* gene structure and pairwise LD between the five tagSNPs.* SIRT6* gene consists of 8 exons (boxes, E1~E8) separated by 7 introns and spans a region of 8,427 bp. Filled boxes indicate the coding regions. The arrows indicate the locations of single nucleotide polymorphism. *D*′ values are plotted as a graph to show linkage disequilibrium between the five tagSNPs.

The Chinese Han population's SNP data of* SIRT6* gene (8,427 bp, 8 exons) plus 3 kb upstream and downstream was obtained in the HapMap data release 27 (http://www.hapmap.org/) [26]. Further analysis of these data was performed utilizing Haploview software version 4.2. Using linkage disequilibrium patterns with *r* ^2^ > 0.8 as a cutoff, five tagSNPs (rs11878868, rs107251, rs352493, rs4807546, and rs3760908) in* SIRT6 *gene were selected for genotyping. All of the tagSNPs have a MAF (minor allele frequency) > 0.05 in the HapMap Chinese Han population. The positions of SNPs were shown in Figure 1. These five tagSNPs would capture the information of 7 known* SIRT6* SNPs with a MAF > 0.05. The *r* ^2^ information for the five tagSNPs and alleles captured accordingly was exhibited in Table S1 (see Supplementary Material available online at http://dx.doi.org/10.1155/2016/1628041). The potential functions of these SNPs were predicted by a platform freely available online (http://snpinfo.niehs.nih.gov/snpinfo/snpfunc.htm). The haplotypic blocks of the five tagSNPs were estimated by the Haploview software version 4.2 and then the haplotype analysis was done by using the SHEsis software (http://analysis.bio-x.cn/myAnalysis.php)[27]. Six common haplotypes (frequency > 3%) derived from the five tagSNPs accounted for almost 100% of the haplotype variations. The allelic sequence in the haplotypes is in the following order: rs11878868, rs107251, rs352493, rs4807546, rs3760908. For example, haplotype GCCTA represents rs11878868G-rs107251C-rs352493C-rs4807546T-rs3760908A.

Genomic DNA was genotyped by PCR-LDR method (Shanghai Biowing Applied Biotechnology Company). The sequences of probes and primers were summarized in Table S2. The PCR was performed on the GeneAmp PCR system 9600 (Perkin Elmer, Norwalk, CT, USA) in 20 *μ*L total volume including 50 ng genomic DNA, 1x PCR buffer, 1 U Taq polymerase, 2 mM dNTPs, 3 mM MgCl_2_, and 0.5 *μ*M primer mix. The cycling parameters were as follows: 95°C for 2 min; 40 cycles at 94°C for 90 s, 56°C for 90 s, and 65°C for 30 s; and a final extension step at 65°C for 10 min. The ligation reaction for each PCR product was performed with a final volume of 10 *μ*L containing 4 *μ*L of PCR product, 1 *μ*L 1x buffer, 2 U Taq DNA ligase, and 2 pmol probe mixture. The LDR parameters were as follows: 95°C for 2 min and 40 cycles at 94°C for 15 s and 50°C for 25 s. Following the LDR reaction, 1 *μ*L LDR reaction product was mixed with 1 *μ*L loading buffer and 1 *μ*L ROX. The mixture was then analyzed by the ABI Prism 3730 DNA Sequencer (Applied Biosystems, USA). About 10% of these samples were randomly selected for repeated genotyping for confirmation, and the results were 100% concordant.

### 2.5. Statistical Analysis

The statistical analyses were performed using the SPSS software package (version 21). The haplotype analysis on the polymorphisms was undertaken by using the SHEsis online webserver. All the five* SIRT6* tagSNPs were tested for confirmation within Hardy-Weinberg expectations through a goodness-of-fit *χ* ^2^ test in the control subjects. Qualitative variables were presented as percentages, and quantitative variables were expressed as means ± standard deviation. Student's *t*-test was used for continuous variables and *χ* ^2^ test was used for categorical variables in the differences of the demographic characteristics between the CAD and control groups. The association of* SIRT6* tagSNPs with CAD severity was evaluated by *χ* ^2^ test. The association of* SIRT6 *polymorphisms with the severity of CAD has been determined by logistic regression model with adjustment by age, sex, smoking, drinking, hypertension, diabetes, and hyperlipidemia. Association between the risk for CAD and the tagSNP was evaluated via logistic regression analysis, adjusted by conventional risk factors (age, sex, drinking, smoking, diabetes, hypertension, and hyperlipidemia). *P* values of less than 0.05 were considered statistically significant for all statistical tests.

## 3. Results

### 3.1. Characteristics of Study Subjects

The baseline characteristics of the case and control subjects were presented in Table 1. Compared with the controls, a higher rate of patients with CAD were male, smoking and alcohol consumers, with prevalence of hypertension, dyslipidemia, and diabetes, and with higher levels of SBP, FBG, TG, and LDL-C, but lower HDL-C. These data demonstrated that male gender, alcohol intake, smoking, hyperlipidemia, hypertension, and diabetes mellitus were the important risk factors for developing CAD in this study.

### 3.2. Multivariate Associations of SIRT6 tagSNPs with the Risk of CAD

Five* SIRT6* tagSNPs (rs11878868, rs107251, rs352493, rs4807546, and rs3760908) were genotyped in 474 CAD patients and 655 control subjects. The primary information for these tagSNPs was shown in Table 2. MAFs for all five tagSNPs in our controls were similar to those for Chinese subjects in HapMap database (Table 2). Genotype frequencies did not deviate from the Hardy-Weinberg equilibrium in the controls (all *P* values ≥ 0.05, Table 2), providing no evidence of population stratification within the dataset. After adjustment for the risk factors including age, gender, drinking, smoking, hypertension, hyperlipidemia, and diabetes, there was no significant difference between CAD cases and controls in the genotype and allele frequencies in these five tagSNPs (all *P* values ≥ 0.05, Table 3).

### 3.3. Association between the Haplotypes of SIRT6 tagSNPs with the Risk of CAD

Haplotype analyses were performed to further investigate the combinational effects of these tagSNPs on CAD risk. As shown in Figure 1, the five tagSNPs were located in one haplotypic block. We thus further assessed the haplotype frequencies of the five tagSNPs between CAD group and controls. Haplotype with frequency over 0.03 will be considered in statistical analysis [28]. Six common haplotypes derived from the five tagSNPs accounted for almost 100% of the haplotype variations. Among the six common haplotypes, no haplotype was found to be associated with the risk for CAD (Table 4).

### 3.4. The Association of SIRT6 tagSNPs with the Severity of CAD

The severity of CAD was evaluated according to the number of vessels with significant stenosis. To analyze a possible association between* SIRT6* tagSNPs and the severity of CAD, patients were grouped into those with 1- and 2-vessel disease and 3-vessel disease (50% luminal obstruction) coronary arteries. The association of* SIRT6 tagSNPs* with the severity of disease was presented in Table 5. By *χ* ^2^ test and logistic regression analysis, we found that rs352493 polymorphism in* SIRT6* exhibited a significant effect on the severity of CAD in the Chinese Han population; C allele (*χ* ^2^ = 7.793, *P* = 0.005, and adjusted *P* = 0.013) and the combined CC/CT genotypes (*χ* ^2^ = 5.609, *P* = 0.018, and adjusted *P* = 0.031) tended to have greater CAD severity. In addition, A allele (*χ* ^2^ = 5.208, *P* = 0.022, and adjusted *P* = 0.046) and AA (*χ* ^2^ = 4.842, *P* = 0.028, and adjusted *P* = 0.054) of rs3760908 also presented the greater CAD severity in the Chinese Han population.

## 4. Discussion

CAD is a complex multifactorial, polygenic disorder that results from both the various environmental factors and the individual's genetic makeup. Recent studies have demonstrated the critical roles of* SIRT6* in DNA damage repair, lipid metabolism, and atherosclerosis, providing evidences that* SIRT6* may play an important role in the CAD pathogenesis [20, 21]. Nonetheless, the association of SNPs in* SIRT6* gene with CAD risk and severity is largely unknown. In the present study, we proceeded with a genetic association analysis on the five* SIRT6* tagSNPs (rs11878868, rs107251, rs352493, rs4807546, and rs3760908) in 474 CAD cases and 655 controls. Our results revealed that C allele of rs352493 and A allele of rs3760908 conferred the increased severity of CAD patients in Chinese subjects, though all the five SNPs and haplotypes showed no significant effect on the risk of CAD. Our study suggested that* SIRT6* rs352493 and rs3760908 SNPs might play roles in the progression of CAD, which could be useful as predictors of the severity of CAD.

The function of* SIRT6* on various physiological and pathological processes has been widely studied, and SIRT6 deficiency or dysregulation is closely associated with diverse human diseases including cardiac hypertrophy, adipocyte disorders, and atherosclerosis [18, 29–31]. Nonetheless, the relationship between* SIRT6* variants and the risk of these diseases remains largely unknown. Previous study has revealed that T-carriers of rs107251 polymorphism in* SIRT6 *exhibited an enhanced risk of carotid plaque [32], while individuals with either CC or CT genotype at rs107251 within* SIRT6* displayed >5-year mean survival advantages compared to TT genotype [33], which is consistent with the protective role of* SIRT6*. Alternatively,* SIRT6* tagSNP rs4807546 showed no significant association with the risk of Parkinson's disease in a Spanish population [34], and the* SIRT6* polymorphisms (rs350852, rs7246235, rs107251, and rs350844) were not associated with diabetic nephropathy in a combined meta-analysis as well [35]. Thus, it was not unexpected that the five tagSNPs (rs11878868, rs107251, rs352493, rs4807546, and rs3760908) in this study exhibited no significant association with the risk of CAD.

Multiple variants or polymorphisms within candidate genes have been reported to be associated with the severity of CAD, which is defined by the number of vessels with significant stenosis [36]. CT or TT genotype of rs7903146 polymorphism in* TCF7L2 *exhibited a higher prevalence and severity of CAD [37]; and the T allele of rs5065 polymorphism in* ANP* showed a decreased severity of CAD in an Iranian population [38]. We herein presented the evidence that C allele of rs352493 and A allele of rs3760908 polymorphisms conferred the increased severity of CAD patients in the Chinese Han population, further expanding the knowledge of the associations between polymorphisms and the progression of CAD. Nonetheless, the underlying molecular mechanisms of* SIRT6* polymorphisms on the severity of CAD still warrant further investigations.

Several limitations in this study still need to be addressed. First, the CAD patients and control subjects enrolled from hospitals may not represent the general population. Nonetheless, the genotype distribution of the control subjects was in Hardy-Weinberg equilibrium. Second, the relatively moderate sample size limited the statistical power of our study. Finally, further studies in different population could help to further verify the significance of the association between the rs352493 and rs3760908 polymorphisms and the severity of CAD. However, our data provided valuable insights into this area.

## 5. Conclusion

In aggregate, our study unraveled that tagSNPs rs352493 and rs3760908 in* SIRT6* gene exhibited a significant effect on the severity of CAD in the Chinese Han population, while all the five tagSNPs and haplotypes showed no significant effect on CAD risk. Further studies with diverse ethnic populations and larger sample size are needed to confirm the general validity of our findings.

## Supplementary Material

The information for alleles captured by rs11878868, rs107251, rs352493, rs4807546 and rs3760908 was shown in TABLE S1. The sequences of the primers and probes used to genotype the rs11878868, rs107251, rs352493, rs4807546 and rs3760908 polymorphisms were shown in TABLE S2.

## Figures and Tables

**Table 1:** The characteristics of CAD cases and controls.

Variable	Controls (*n* = 655)	Cases (*n* = 474)	*P* ^a^
Age (years)	61.50 ± 12.50	63.19 ± 11.68	**0.021**
Sex (male)	379 (57.9%)	362 (76.4%)	**<0.001** ^b^
Smoking	154 (23.5%)	286 (60.3%)	**<0.001**
Alcohol consumption	99 (15.1%)	123 (25.9%)	**<0.001**
Hypertension	221 (33.7%)	293 (61.8%)	**<0.001**
Diabetes	104 (15.9%)	229 (48.3%)	**<0.001**
Hyperlipidemia	239 (36.5%)	314 (66.2%)	**<0.001**
Systolic BP (mm Hg)	131.83 ± 19.38	139.70 ± 19.80	**<0.001**
Diastolic BP (mm Hg)	72.78 ± 10.63	73.42 ± 11.24	0.332
FPG (mM)	5.79 ± 1.95	6.27 ± 1.64	**<0.001**
Triglycerides (mM)	1.48 ± 0.81	1.90 ± 1.09	**<0.001**
Total cholesterol (mM)	4.59 ± 1.15	4.67 ± 1.21	0.266
HDL cholesterol (mM)	1.39 ± 0.67	1.24 ± 0.44	**<0.001**
LDL cholesterol (mM)	2.61 ± 0.92	2.88 ± 0.92	**<0.001**

^a^Two-sided chi-square test or independent-samples *t*-test.

^b^ *P* values under 0.05 were indicated in bold font.

**Table 2:** Primary information for tagSNPs in *SIRT6 *gene.

Genotyped SNPs	rs11878868	rs107251	rs352493	rs4807546	rs3760908
Chr Pos (genome build 106)	4173640	4176088	4180839	4182063	4184515
Pos in *SIRT6* gene	3′ UTR	Intron 4	Intron 2	Intron 1	5′ UTR
MAF^a^ for Chinese (CHB) population in HapMap	0.110	0.310	0.240	0.390	0.430
MAF in controls (*n* = 655)	0.097	0.296	0.254	0.365	0.434
*P* value for HWE^b^ test in controls	0.411	0.773	0.785	0.839	0.093

^a^MAF: minor allele frequency.

^b^HWE: Hardy-Weinberg equilibrium.

**Table 3:** Multivariate associations of tagSNPs in *SIRT6* gene with the risk of CAD.

Type	Controls (*n* = 655)	Cases (*n* = 474)	OR (95% CI)^a^	*P* ^a^
	Number (%)	Number (%)		
rs11878868				
Additive				
G	1183 (90.3)	856 (90.3)	1	
T	127 (9.7)	92 (9.7)	0.97 (0.69–1.36)	0.870
Dominant				
GT + GG	647 (98.8)	471 (99.4)	1	
TT	8 (1.2)	3 (0.6)	0.60 (0.14–2.57)	0.487
Recessive				
GG	536 (81.8)	385 (81.2)	1	
GT + TT	119 (18.2)	89 (18.8)	1.00 (0.69–1.45)	0.993

rs107251				
Additive				
T	388 (29.6)	279 (29.4)	1	
C	922 (70.4)	669 (70.6)	1.13 (0.90–1.41)	0.293
Dominant				
TT	59 (9.0)	37 (7.8)	1	
CC + CT	596 (91.0)	437 (92.2)	1.65 (0.98–2.76)	0.060
Recessive				
CT + TT	329 (50.2)	242 (51.1)	1	
CC	326 (49.8)	232 (48.9)	1.04 (0.78–1.39)	0.774

rs352493				
Additive				
T	977 (74.6)	721 (76.1)	1	
C	333 (25.4)	227 (23.9)	0.99 (0.78–1.25)	0.921
Dominant				
CC	41 (6.3)	30 (6.3)	1	
CT + TT	614 (93.7)	444 (93.7)	0.98 (0.54–1.78)	0.944
Recessive				
CT + CC	292 (44.6)	197 (41.6)	1	
TT	363 (55.4)	277 (58.4)	0.99 (0.74–1.32)	0.929

rs4807546				
Additive				
C	478 (36.5)	352 (37.1)	1	
T	832 (63.5)	596 (62.9)	0.94 (0.76–1.16)	0.565
Dominant				
TT	263 (40.2)	189 (39.9)	1	
CT + CC	392 (59.8)	285 (60.1)	0.96 (0.72–1.28)	0.763
Recessive				
CT + TT	569 (86.9)	407 (85.9)	1	
CC	86 (13.1)	67 (14.1)	0.86 (0.57–1.30)	0.478

rs3760908				
Additive				
G	741 (56.6)	562 (59.3)	1	
A	569 (43.4)	386 (40.7)	0.95 (0.77–1.17)	0.616
Dominant				
AA	113 (17.3)	86 (18.1)	1	
AG + GG	542 (82.7)	388 (81.9)	0.88 (0.60–1.29)	0.506
Recessive				
GG	199 (30.4)	174 (36.7)	1	
AG + AA	456 (69.6)	300 (63.3)	0.83 (0.61–1.12)	0.212

^a^Adjusted for age, sex, smoking, drinking, hypertension, diabetes, and hyperlipidemia.

**Table 4:** Association between haplotypes of tagSNPs in *SIRT6 *gene with the risk of CAD.

Haplotype^a^	Controls	Cases	OR (95% CI)	*P*
	Number (%)	Number (%)		
Total	*n* = 655	*n* = 474		
GCCTA	328.69 (25.1)	223.42 (23.6)	0.93 (0.76–1.12)	0.432
GCTCG	86.43 (6.6)	71.83 (7.6)	1.17 (0.84–1.61)	0.356
GCTTA	108.25 (8.3)	62.54 (6.6)	0.79 (0.57–1.09)	0.145
GCTTG	266.76 (20.4)	214.82 (22.7)	1.15 (0.94–1.41)	0.174
GTTCG	385.25 (29.4)	274.28 (28.9)	0.98 (0.82–1.18)	0.846
TCTTA	125.02 (9.5)	90.91 (9.6)	1.01 (0.76–1.34)	0.950

^a^The allelic sequence in the haplotypes is in the following order: rs11878868, rs107251, rs352493, rs4807546, rs3760908.

**Table 5:** *SIRT6* tagSNPs in association with diseased vessel (*n* = 474).

Type	1VD and 2VD^a^ (*n* = 292)	3VD (*n* = 182)	*χ* ^2^	*P*	OR (95% CI)^c^	*P* ^c^
	Number (%)	Number (%)				
rs11878868						
G	530 (90.8)	326 (89.6)	0.364	0.546	1	0.563
T	54 (9.2)	38 (10.4)			1.14 (0.73–1.80)	
GG	240 (82.2)	145 (79.7)	0.467	0.494	1	0.501
GT + TT	52 (17.8)	37 (20.3)			1.18 (0.73–1.90)	

rs107251						
C	405 (69.3)	264 (72.5)	1.091	0.296	1	0.463
T	179 (30.7)	100 (27.5)			0.89 (0.66–1.21)	
CC	139 (47.6)	93 (51.1)	0.548	0.459	1	0.647
CT + TT	153 (52.4)	89 (48.9)			0.92 (0.63–1.34)	

rs352493						
T	462 (79.1)	259 (71.2)	7.793	** 0.005** ^b^	1	**0.013**
C	122 (20.9)	105 (28.8)			1.47 (1.09–2.00)	
TT	183 (62.7)	94 (51.6)	5.609	**0.018**	1	**0.031**
CC + CT	109 (37.3)	88 (48.4)			1.52 (1.04–2.24)	

rs4807546						
T	360 (61.6)	236 (64.8)	0.978	0.232	1	0.443
C	224 (38.4)	128 (35.2)			0.90 (0.68–1.18)	
TT	109 (37.3)	80 (44.0)	2.054	0.152	1	0.177
CT + CC	183 (62.7)	102 (56.0)			0.77 (0.52–1.13)	

rs3760908						
A	221 (37.8)	165 (45.3)	5.208	**0.022**	1	**0.046**
G	363 (62.2)	199 (54.7)			0.76 (0.59–1.00)	
AA	44 (15.1)	42 (23.1)	4.842	**0.028**	1	0.054
GG + AG	248 (84.9)	140 (76.9)			0.62 (0.38–1.01)	

^a^1VD: 1-vessel disease; 2VD: 2-vessel disease; 3VD: 3-vessel disease. ^b^ *P* values under 0.05 were indicated in bold font. The *P* ^c^ values and OR^c^ were adjusted for age, sex, smoking, drinking, hypertension, diabetes, and hyperlipidemia.
