# Supplementary material for: Two tagSNPs rs352493 and rs3760908 within SIRT6 Gene Are Associated with the Severity of Coronary Artery Disease in a Chinese Han Population
Source: Dis Markers. 2016 Mar 28;2016:1628041. doi: 10.1155/2016/1628041 (PMC4826929; doi:10.1155/2016/1628041)
Supplement: Supplementary file 1 — The information for alleles captured by rs11878868, rs107251, rs352493, rs4807546 and rs3760908 was shown in TABLE S1. The sequences of the primers and probes used to genotype the rs11878868, rs107251, rs352493, rs4807546 and rs3760908 polymorphisms were shown in TABLE S2. [file 1628041.f1.pdf]

TABLE S1: The information for alleles captured by rs11878868, rs107251, rs352493, rs4807546 and rs3760908, accordingly.

Alleles captured	Location	tagSNP	r^2	Potential function [a]		Reported traits	Reference
				TFBS	miRNA		
rs11878868	3' UTR	rs11878868	1.0	-	-	-	-
rs350844	Intron 4	rs107251	1.0	-	-	-	-
rs107251	Intron 4	rs107251	1.0	-	-	carotid plaque, Aging and longevity	[31,32]
rs352493	Intron 2	rs352493	1.0	-	-	-	-
rs4807546	Intron 1	rs4807546	1.0	Y	-	-	-
rs3760908	5' UTR	rs3760908	1.0	Y	-	-	-
rs3760905	5' UTR	rs3760908	1.0	Y	-	carotid plaque	[31]

[a] The potential functions of the SNPs were predicted by online webserver (http://snpinfo. niehs.nih.gov/snpinfo/snpfunc.htm)

TABLE S2: The sequences of the primers and probes used to genotype the rs11878868, rs107251, rs352493, rs4807546 and rs3760908 polymorphisms.

Name	Sequence (5’-3’)
**Primers**	
rs11878868-F	AGGAACAAGGGCTCACCTCT
rs11878868-R	GCTGCCTTATTGGCTTCATC
rs107251-F	TGACCAGAACTAGCACCCAG
rs107251-R	GCTTTGTTTTCTGTGCTCCG
rs352493-F	ATGCCAGAGGCAGTGCTGAT
rs352493-R	AAGGTGTGGGAACTGGCGA
rs4807546-F	CTTTATCCTGAGGGTGATGG
rs4807546-R	TGGCTGGAGGGATCTTTCTG
rs3760908-F	ACACACATGCACTCGTTCAC
rs3760908-R	TGATGGCCGGGTGGGATGTT
**Probes**	
rs11878868_modify	P-TCTGGAGGGATCAGGCCAGTTTTTTTTTTTTTTTTTTTTT-FAM
rs11878868_G	TTTTTTTTTTTTTTTTTTCTCACCTCTCCCCCCACCCCGCC
rs11878868_T	TTTTTTTTTTTTTTTTTTTTCTCACCTCTCCCCCCACCCCGCA
rs107251_modify	P-TGGAAGGCTACTCGTTCCGTTTTTTTTTTTTTTTTTTTTTTTTT-FAM
rs107251_C	TTTTTTTTTTTTTTTTTTTTTTGCAGCATCCCAGCCCGTTATTCG
rs107251_T	TTTTTTTTTTTTTTTTTTTTTTTTGCAGCATCCCAGCCCGTTATTCA
rs352493_modify	P-TGTGGTGTTCCACACGGGTGTTTTTTTTTTTTTTTTTTTTTTTTTT-FAM
rs352493_C	TTTTTTTTTTTTTTTTTTTTTTTTAGGCTGGTCTGGCAGTCTTCCAG
rs352493_T	TTTTTTTTTTTTTTTTTTTTTTTTTTAGGCTGGTCTGGCAGTCTTCCAA
rs4807546_modify	P-ATGTGCCAGAAAGATCCCTCTTTTTTTTTTTTTTTTTTTTTTTTTTTT-FAM
rs4807546_C	TTTTTTTTTTTTTTTTTTTTTTTTTTCCCTGGCGAGGGGACTGGACAGG
rs4807546_T	TTTTTTTTTTTTTTTTTTTTTTTTTTTTCCCTGGCGAGGGGACTGGACAGA
rs3760908_modify	P-GCCTTGGAGGAAGCTAGGCATTTTTTTTTTTTTTTTTTTTTTTTTTTTTTTT-FAM
rs3760908_A	TTTTTTTTTTTTTTTTTTTTTTTTTTTTTTGCCGGGTGGGATGTTGAGCACCT
rs3760908_G	TTTTTTTTTTTTTTTTTTTTTTTTTTTTTTTTGCCGGGTGGGATGTTGAGCACCC
